# A Literature Review of Traumatic Brain Injury Biomarkers

**DOI:** 10.1007/s12035-022-02822-6

**Published:** 2022-04-30

**Authors:** Hazem S. Ghaith, Asmaa Ahmed Nawar, Mohamed Diaa Gabra, Mohamed Essam Abdelrahman, Mohamed H. Nafady, Eshak I. Bahbah, Mahmoud Ahmed Ebada, Ghulam Md Ashraf, Ahmed Negida, George E. Barreto

**Affiliations:** 1 Faculty of Medicine, Al Azhar University, Cairo, Egypt; 2 Faculty of Medicine, Zagazig University, Zagazig, Egypt; 3 Faculty of Medicine, South Valley University, Qena, Egypt; 4 Faculty of Applied Medical Science, Misr University for Science and Technology, First 6th of October, Egypt; 5 Faculty of Medicine, Al-Azhar University, New Damietta, Egypt; 6 Ministry of Health and Population Hospitals, Cairo, Egypt; 7 Pre-clinical Research Unit, King Fahd Medical Research Center, King Abdulaziz University, Jeddah, Saudi Arabia; 8 Department of Medical Laboratory Technology, Faculty of Applied Medical Sciences, King Abdulaziz University, Jeddah, Saudi Arabia; 9 School of Pharmacy and Biomedical Sciences, University of Portsmouth, Portsmouth, UK; 10 Department of Biological Sciences, University of Limerick, Limerick, Ireland

**Keywords:** Biomarkers, Exosomes, Head injury, miRNA, Traumatic brain injury

## Abstract

Research into TBI biomarkers has accelerated rapidly in the past decade owing to the heterogeneous nature of TBI pathologies and management, which pose challenges to TBI evaluation, management, and prognosis. TBI biomarker proteins resulting from axonal, neuronal, or glial cell injuries are widely used and have been extensively studied. However, they might not pass the blood-brain barrier with sufficient amounts to be detected in peripheral blood specimens, and further might not be detectable in the cerebrospinal fluid owing to flow limitations triggered by the injury itself. Despite the advances in TBI research, there is an unmet clinical need to develop and identify novel TBI biomarkers that entirely correlate with TBI pathologies on the molecular level, including mild TBI, and further enable physicians to predict patient outcomes and allow researchers to test neuroprotective agents to limit the extents of injury. Although the extracellular vesicles have been identified and studied long ago, they have recently been revisited and repurposed as potential TBI biomarkers that overcome the many limitations of the traditional blood and CSF assays. Animal and human experiments demonstrated the accuracy of several types of exosomes and miRNAs in detecting mild, moderate, and severe TBI. In this paper, we provide a comprehensive review of the traditional TBI biomarkers that are helpful in clinical practice. Also, we highlight the emerging roles of exosomes and miRNA being the promising candidates under investigation of current research.

## Introduction

Traumatic brain injury (TBI) is one of the leading causes of disability and death worldwide [1]. TBI affects approximately 50 million individuals annually [2]. It can result from exposure to a blow or blast, rapid head deceleration or acceleration, and skull penetration [3]. These factors may lead to internal hemorrhage, bruises, lacerations, focal and diffuse injuries, hypoxia, and interference in axonal connectivity [4]. TBI may also give rise to many neurodegenerative disorders and increase survivors’ risk of developing chronic behavioral and neurological impairment that affects the quality of their lives. Factors underlying the susceptibility of individuals to develop these disorders are still intensively unknown.

TBI can cause alteration in cognition and attention, anxiety, aggression, depression, and personality changes. It is also believed to raise the risk of developing some diseases, including Parkinson’s disease and Alzheimer’s disease [5]. According to the Glasgow Coma Scale Score (GCS), TBI severity can be classified into mild, moderate, and severe injuries [6]. TBI severity can be determined by the location and nature of the injury and the occurrence of complications such as elevated intracranial pressure [7]. The pathophysiology of TBI was reported to include the release of cytokines, chemical mediators, and many neurotransmitters that contribute to the process of tissue injury. Also, nitric oxide and calcium both play a significant role in apoptosis induction [8]. TBI-heterogeneous nature has limited the complete understanding of its pathophysiology, and this has mainly been reflected in the development of effective therapeutic strategies. Therefore, there is an urgent need for better diagnostic modalities for TBI.

Brain injuries cause a yearly economic burden, encouraging researchers to find effective, safe, and cheap TBI monitoring methods [3]. Some studies have investigated some molecules released in the body fluids due to pathological mechanisms of TBI [9]. Of these biofluid biomarkers, extracellular vesicles (EVs) and miRNAs have been reported to be promising biomarkers for TBI [9]. Recently, miRNAs and EVs have gained significant attention in the scientific community for their actions in many pathological and physiological processes. In this article, we provide a comprehensive review of the traditional TBI biomarkers that are helpful in clinical practice. Also, we highlight the emerging roles of exosomes and miRNA being the promising candidates under investigation of current research.

## The Urgent Need for More TBI Biomarkers

TBI is not a single event, but it includes complex pathological processes. TBI may involve a primary injury that arises immediately after the trauma and secondary injuries resulting in a series of molecular and cellular reactions that last for a long time after the trauma, leading to neuronal and astroglia injuries, axonal disruption, and inflammation. A comprehensive understanding of TBI pathophysiological processes is essential to develop neuroprotective and therapeutic interventions [10]. Integration of biomarkers, clinical examination, and imaging techniques can lead to proper severity detection, outcome prediction, and evaluation of response to clinical management after TBI [11]. TBI biomarkers are needed to diagnose TBI and advance neuroprotective and therapeutic clinical trials. The lack of reliable biomarkers that entirely correlate with the pathogenesis and progression of TBI leads to undiagnosed or late-diagnosed disease.

Mild TBI patients mostly show no symptoms for days or weeks after injury. Neuroimaging techniques such as computed tomography (CT), imaging spectroscopy, and magnetic resonance imaging (MRI) help evaluate gross head injuries but not mild TBI since imaging cannot detect minute neural and structural changes [12, 13]. Besides, MRI may be unavailable in clinical settings or costly for some patients [14]. On the other hand, fluid biomarkers are more accurate tools for assessing TBI pathophysiology. Current conventional fluid biomarkers of TBI are limited as they exist in minimal concentrations; therefore, they require sensitive assays. Furthermore, they might be undetectable in many cases owing to the limitations in their diffusion across the blood-brain barrier in some patients and further challenged by the fluid disturbance and cerebrospinal fluid (CSF) flow limitations inflicted by the brain injury. Therefore, there is an unmet clinical need to find fluid biomarkers to detect all TBIs; assess the severity of TBIs, including mild TBI; and predict the risk of developing long-term consequences such as post-concussive syndrome and chronic traumatic encephalopathy [15].

According to Wang et al. [10], the ideal and reliable fluid biomarker of TBI should meet the following criteria: (1) First, head trauma should induce the release of that biomarker into accessible body fluids; (2) the biomarker level must increase significantly in TBI patients than in healthy people; (3) the biomarker should correlate quantitatively and qualitatively with injury severity; and (4) the level of the biomarker should be associated with other clinical diagnostic tools as GCS, CT, and MRI [10].

## Traditional Techniques of TBI Biomarker Measurement

### Enzyme-Linked Immunosorbent Assay

Immunochemical techniques usually assess TBI biomarkers. Enzyme-linked immunosorbent assay (ELISA) is the most frequently used test, especially sandwich ELISA. In sandwich ELISA, the antigen (the biomarker of interest) reacts with two types of antibodies, capturing and detection antibodies. Antibody detection produces a color change that indicates the presence of the antigen. In addition to this technique, another one is multiplex ELISA that can react with more than one antigen simultaneously in a single sample**.** Multiplex ELISA provides a comprehensive and accurate quantification of the biomarkers. ELISA sensitivity relies on the quality of the used antibody. When the antibody cross-reacts with other antigens, the test will increase the biomarker concentration than the real one. Blood samples commonly show increased biomarkers incorrectly because blood contains many plasma proteins [15].

### Mass Spectrometry

Mass spectrometry (MS) is a common technique that assesses proteins in an antibody-independent manner [15, 16]. It is used in laboratory examinations to detect small molecules like hormones, vitamins, and drugs. MS also measures proteins that indicate neural degeneration after TBI, which is used in preclinical studies to discover new TBI biomarkers [16]. It measures biomarkers with high sensitivity, making it an ideal diagnostic and prognostic modality [11].

## Types of TBI Biomarkers

**Fig. 1:**
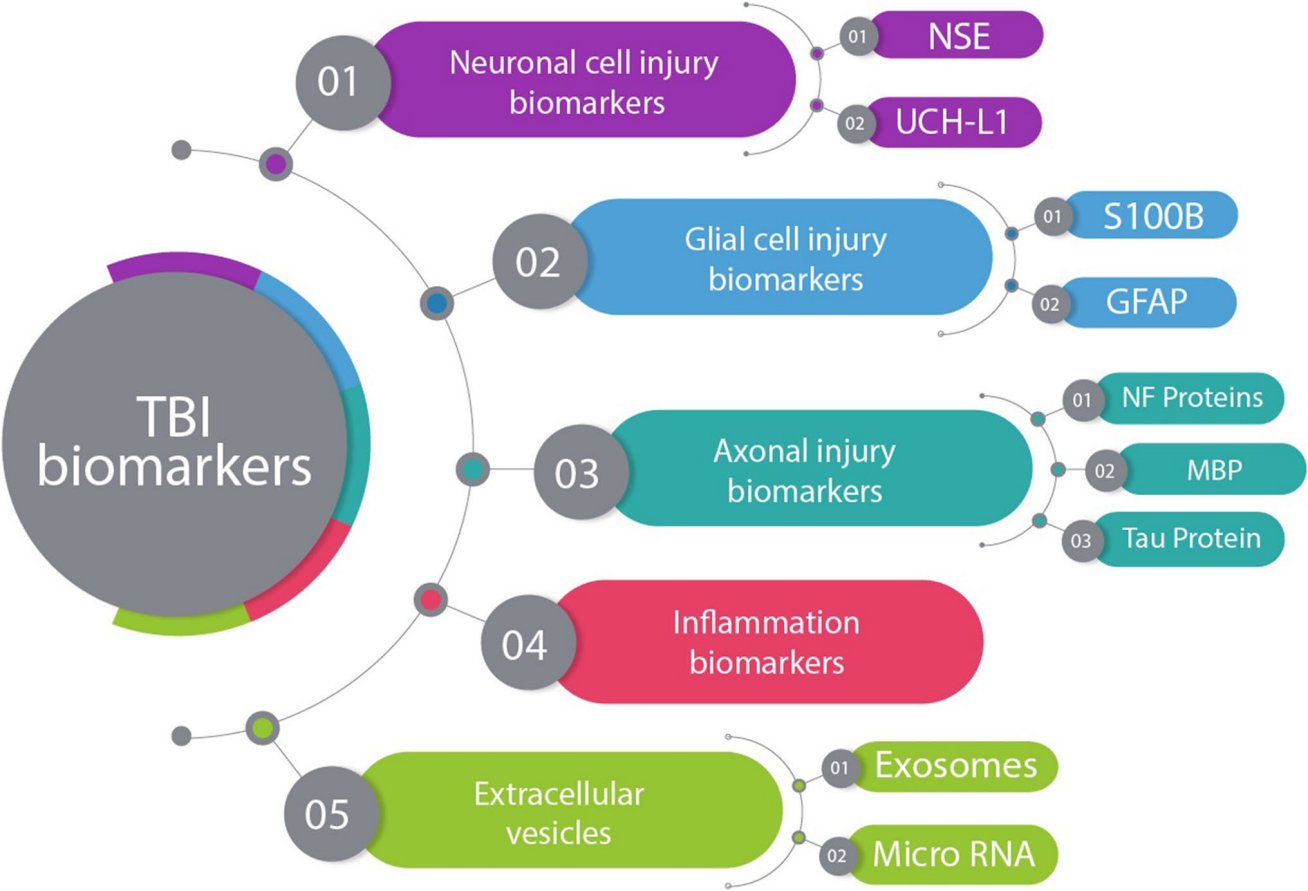
Flow diagram of the different types of TBI biomarkers that denote many processes such as neuronal injury, glial injury, axonal injury, and inflammation. NSE: neuron-specific enolase; UCH-L1: ubiquitin C-terminal hydrolase-L1; GFAP: glial fibrillary acidic protein; and MBP: myelin basic protein

Research in the past decades revealed several molecules that can mirror the changes and the pathophysiological reactions of TBI. These molecules act as potential biomarkers and denote processes such as neuronal injury, glial injury, axonal injury, and inflammation (Fig. 1). We summarized the potential biomarkers, their targets, and their association with different pathologies and severities of TBI (Table 1).

**Table 1:** Candidate biomarkers associated with TBI severity and the pathological mechanisms

Event	Mechanisms	Candidate biomarkers		
		Mild TBI	Moderate TBI	Severe TBI
Neuronal and glial cell injury	Activation of factors triggering necrosis and/or apoptosis	Neuronal: NSE, C- tau, all-spectrin	Neuronal: NSE, UCH-L-1, PNF-H, NMDAR, all-spectrin, C-tau, Hsp70	Neuronal: NSE, UCH-1, C-tau, Hsp70, all-spectrin, NMDAR
		Glial: S100B, GFAP, MBP, C-tau	Glial: S100B, NMDAR, MBP, IL-1B, GFAP, C-tau, IL-6, IL-8	Glial: S100B, GFAP, IL-6, IL-8, MBP, AQP4
Axonal injury	Mechanical injury; neuronal degeneration	S100B, NSE, C-tau, MBP, SBP, all-spectrin	S100b, MBP, NSE, C-tau, PNF-H, Hsp70, SBP	S100B, MBP, NSE, PNF-H, C-tau, Hsp70, SBP
Inflammation	Cytokine release and cellular stress	IL-1B, IL-6, IL-8, TNF-α, IFN-γ	Hsp70, IL-1B, IL-6, IL8, TNF-α, IFN-γ	Hsp70, IL-1B, IL-6, IL8, TNF-α, IFN-γ

### Neuronal Cell Body Injury

#### Neuron-Specific Enolase

Neuron-specific enolase **(**NSE) is a neuronal cytoplasmic enzyme that plays a role in the glycolytic pathway of nerve cells. In this regard, extracellular NSE indicates neuron cell injury [17]. Serum NSE concentration rises in the first 12 h after TBI and declines within hours or days. Patients with secondary NSE increase exhibit more severe outcomes. Moderate and severe TBI patients, who suffer from persistently elevated serum NSE levels, are significantly at risk of poor neurological effects and mortality [18, 19]. Serum NSE usually increases in patients with a GCS score ≤ 3 [19]. The main drawback of using NSE as a TBI diagnostic tool is its high erythrocyte concentrations. Therefore, hemolysis increases serum NSE, even without the coexistence of any TBI [18].

#### Ubiquitin C-Terminal Hydrolase-L1

Ubiquitin C-terminal hydrolase-L1 **(**UCH-L1) is an enzymatic protein in the cytoplasm nerve cells. UCH-L1 contributes to removing abnormal neuronal proteins in both physiological and pathological conditions [11, 20]. Studies evaluated serum and CSF UCH-L1 concentration elevation within the first 6–24 h after TBI [21]. Increased serum UCH-L1 is attributed to the breakdown of BBB after moderate to severe TBI [22–24]. Serum UCH-L1 concentration after TBI correlates with injury severity and clinical outcomes, including GCS score at the time of admission and lesions on CT [25, 26]. Within 3 months after severe TBI, mortality is associated with elevated serum and CSF concentration of UCH-L1 in the first 6 h after injury [21]. Finally, UCH-L1 is a potential prognostic and diagnostic biomarker for mild, moderate, and severe TBIs.

### Glial Cell Injury Biomarkers

#### S100B Protein

S100B is a calcium-binding protein present in astroglial cells [10]. Brain trauma and ischemic injuries activate astroglial cells [27–32], which release the S100B protein into the extracellular space. S100B proteins diffuse into CSF and then to blood [33]. Although S100B proteins protect the neurons against post-TBI inflammation, they increase the phosphorylation of tau proteins that cause neurodegeneration [33, 34]. Several studies link elevated S100B protein levels with increased injury severity, poor clinical outcomes [11, 35], and increased mortality [36]. Similarly, S100B protein level can predict the occurrence of the post-concussion syndrome after mild TBI [10, 36]. There is also a positive association between S100 protein levels and intracranial pressure and cranial CT after TBI [36].

S100B protein levels can predict normal CT after mTBI, reducing unnecessary CT scans. S100B protein is currently used in the early control of minimal, mild, and moderate TBI in Scandinavia according to their head injury management guidelines in adults [37]. Although many studies recommend S100B as a prognostic biomarker after TBI, other studies reported a weak prediction tool, especially with long-term outcomes [33, 35, 38]. Another limitation is the extra-neural release of S100B protein from cardiac muscle, adipose tissue, and skeletal muscles [10, 39].

#### Glial Fibrillary Acidic Protein

Glial fibrillary acidic protein (GFAP) is an intermediate filament in astroglial cells [10, 33]. GFAP also exists in non-myelinating Schwann cells in the peripheral nervous system and enteric glial cells. GFAP supports the neurons, maintains their structure, and helps in activating glial cells [11]. Astroglial cells are activated after TBI to induce gliosis or glial scar formation, so the expression of GFAP increases [31, 32, 40–46].

TBI exacerbates the release of GFAP or its breakdown products (GFAP-BDPs) in CSF and blood [47]. Studies show a positive correlation between GFAP levels and TBI severity [26] [11]. GFAP can assess mTBI and evaluate the need for neuroimaging as CT and MRI, which will reduce unnecessary CT and MRI. Thus, GFAP may identify the need for intensive monitoring and predict poor outcomes and the risk for developing cognitive and psychiatric disabilities [14, 26].

### Axonal Injury Biomarkers

#### Neurofilament Proteins

Neurofilament proteins (NFs) are specific neuronal and axonal proteins. NFs are the primary component of the neuronal cytoskeleton to provide integrity and mechanical support. NFs consist of three different polypeptide subunits: a light subunit (NF-L, 68 kDa), a medium subunit (NF-M, 160 kDa), and a heavy subunit (NF-H, 200 kDa) [48, 49]. NFs undergo phosphorylation. Phosphorylated filaments interact with each other to increase neuronal stability [49, 50]. After TBI, intracellular calcium increases and activates various calcium-dependent enzymes like proteases, calpains, and phosphatase calcineurin. These enzymes act on NFs and lead to their dephosphorylation, proteolysis, and dissociation. NF-L, NF-M, and NF-H are released into extracellular space, then to CSF and blood [48, 50]. Due to NF specificity for neurons and axons, their extra-neural detection indicates neural death and axonal disintegration [50].

NF release lasts for days after the trauma, which may predict the occurrence of chronic morbidities and cognitive disability [10]. Studies reported that NF-L and NF-H increase in the first 2 weeks after severe TBI, indicating poor outcomes [51]. Serum NF-L levels correlate with CT lesions. Initial NF-L levels can predict the outcomes 12 months after TBI [49, 52]. NF-L concentration is a sensitive and reliable biomarker in assessing sports-related concussions [53]. Serum phosphorylated NF-H is also a specific biomarker for axonal injury, especially white matter axons [54]. PNF-H levels are associated with injury severity and can predict mortality [11].

#### Myelin Basic Protein

Myelin basic protein (MBP) is the second major protein in the CNS, and it does exist in oligodendroglia. TBI causes axonal damage and the release of MBP in the blood. However, its release occurs 1–3 days after the injury [55, 56]. Elevated serum MBP levels may last for 2 weeks indicating poor outcome, especially intracranial hemorrhage (ICH) [57]. There is an association between increased MBP levels and the increased risk of mortality [58, 59].

Because MBP release is delayed and its initial levels do not correlate with the GCS [58], it is not suitable for the emergency room screening biomarker. MBP is not specific for CNS injury, as injury in the peripheral nerves also increases MBP blood concentration. The presence of both peripheral nerve damage and TBI makes MBP an inaccurate diagnostic and prognostic biomarker [56].

#### Tau Protein

Tau is a microtubule-associated protein (MAP) expressed mainly in the neurons to stabilize axonal microtubules [10, 11]. Normally, tau undergoes phosphorylation to control microtubules’ movement. When this phosphorylation is abnormally excessive, tau accumulates and forms neurofibrillary tangles. These tangles impair neuronal functions and cause neurodegenerative diseases [60]. TBI–related cerebral ischemia induces tau hyperphosphorylation [61], suggesting TBI contributes to chronic neurodegeneration and cognitive impairment [56].

TBI increases tau release in CSF. CSF tau concentration positively correlates with TBI severity and poor outcomes [62, 63]. Previously, Wang et al. demonstrated that serum tau protein peaks 2 days after TBI, while serum tau level reflects injury severity and predicts the clinical outcome [61]. CSF tau is a more accurate diagnostic and prognostic tool than serum tau. Some studies reported that serum tau does not correlate with CT lesions and cannot predict post-concussion syndrome [64].

### Inflammation Biomarkers

Inflammatory biomarker measurement after TBI could potentially help in disease progression monitoring, injury diagnosis, and prediction of long-term outcomes. The inflammatory blood biomarkers could be elevated in response to any disease-causing cellular injury, so they are not highly specific for TBI. However, few studies have reported that these biomarkers could possess a potential clinical utility for TBI patients [65]. Both primary and secondary TBIs stimulate inflammatory processes via activation of inflammatory proteins, mainly cytokines released from activated microglia and leukocytes [66]. These cytokines are elevated after TBI and include interleukin (IL)-1, IL-6, IL-8, IL-10, and TNF-α. The prolonged release of these cytokines contributes to neurodegenerative diseases [67]. Previously, serum IL-1 beta level has been found correlated with injury severity, GCS, and prognosis [68]. IL6 levels in blood increase after TBI and can evaluate injury severity, but it is a poor outcome predictor [66]. Other cytokines IL-8, IL-10, and TNF-α are excessively produced after TBI, but their correlation with injury severity and outcomes is yet to be confirmed [11, 66]. Moderate and severe cerebral hypoperfusion was associated with high levels of IL-8 in the serum of TBI patients [69]. Because biofluid biomarkers of TBI undergo rapid updates, it is extremely difficult to assess a reliable biomarker.

Previously, adiponectin, a marker of inflammation, was reported to be elevated in the plasma of TBI patients and was found to be an independent predictor of unfavorable outcomes and mortality. Also, high-mobility group box 1 (HMGB1), a marker of inflammation and a cytokine, was an important predictor for 1-year mortality in TBI patients. HMGB1 works by translocation to the cytoplasm from the nucleus in early TBI. Following that, it enters phagocytic microglia. According to these findings, HMGB1 may serve as a promising therapeutic target for TBI patients in addition to serving as a biomarker [70–72].

Galectin-3 is a lectin family member which was reported to be also involved in microglial activation. Recent studies have shown that galectin-3 concentrations increase in the plasma of TBI patients. Also, it may play a role as a hospital mortality indicator. On the other hand, poorer outcomes may be associated with lower plasma levels of Ficolin 3, which is a lectin complement pathway activator [73, 74]. In addition, elevated levels of mannose-binding lectins (MBL) have been noticed in the serum of TBI patients [75].

### Extracellular Vesicles

EVs are membranous particles secreted by different cells into body fluids and take part in intercellular communication [76]. EVs are secreted from all types of brain cells. In general, they are lined by a lipid bilayer and contain specific markers on their surface and intraluminal DNA, RNA, protein, and metabolites which act as indicators for the state of glial cells and neurons. They are involved in pathology and recovery processes during CNS injuries [77–79].

EVs are classified into three subtypes, apoptotic cells, exosomes, and MVs [76]. Apoptotic cells are secreted during apoptosis by separating the plasma membrane of the dying cell from the cytoskeleton because of the elevated hydrostatic pressure. They are large, with a size range between 50 and 5000 nm and characterized by containing dying cells’ DNA [80, 81]. The second subtype is called MVs or MPs, with size ranging between 100 and 1000 nm. They are developed through the extracellular budding of the plasma membrane of the cells [80]. Several enzymes and calcium signaling play a role in inducing this outward budding [82]. The third subtype of EVs is called exosome, cup-like shapes with a size range from 30 to 120 nm^2^. They are produced by forming intraluminal vesicles inside intracellular multivesicular bodies by the intraluminal budding of endosomal compartments. These intraluminal vesicles are secreted as exosomes extracellularly through the fusion of bodies with the parent cell plasma membrane [82]. Exosome structures contain non-coding RNAs, proteins, and mRNAs. These components are protected from degradation by the lipid bilayer of exosomes [83]. Exosomes and miRNAs are gaining much attention recently as promising biomarkers for TBI.

EVs are essential biomarkers for the diagnosis, prognosis, and treatment of malignancy and different diseases such as TBI, which makes competent isolation a big challenge for scientists and researchers. Exosome isolation is either from raw biological fluids or conditioned cell culture. Specific components of biological fluids, including lipoproteins, chylomicrons, and microvesicles, interfere with exosome isolation, making isolating exosomes from these fluids very challenging. On the other hand, isolation from conditioned cell culture is not as difficult as in raw biological fluid [84, 85]. There are two sub-groups of isolation techniques, conventional and microfluidics approaches. Although the traditional approach does not demonstrate high efficiency, it is the most used method [86].

Filtration is efficiently sized based on extracellular isolation using a membrane with a suitable pore to filtrate EVs [87]. There is a new effective and flexible technique based on the size of EVs that separates EVs from a colloidal mixture using a permeable membrane subjected to a tangential flow called tangential flow filtration [88]. There are many other size-based methods like size exclusion chromatography and polymer precipitation [89].

Because of their nanoscale size, extracellular isolation faces many difficulties, as it takes more than 6 h in size-based extracellular isolation, so it is a time-consuming method. Also, it contributes to cellular debris co-purification and does not selectively separate particular sub-types; however, the inadequacy in biological sample magnetism makes immunomagnetic sorting facing huge success in selective and effective EV isolation [90–92].

Microfluidics provide a method like the micron-sized channel to filter small volumes of fluids (microliter to picolitre) [93, 94]. The microfluidic-based method can be classified into two groups, the active method, which is characterized by an external force, while the second is called the passive method, which uses hydrodynamic and surface forces [95].

EVs can be detected normally in healthy people. However, EV levels increase in some pathological conditions like TBI [23]. It was found that EVs in the plasma of TBI patients suffering from alterations in consciousness have GFAP ten times more than normal individuals or conscious patients [55]. Also, a difference in miRNA expression in EVs was detected between TBI patients (especially with altered consciousness) and normal individuals. All of these indicate that EVs can be promising biomarkers for TB (Table 2) [55].

**Table 2:** Studies discussing the role of miRNAs and EVs in TBI

Study ID	Type	Biomarker	Key findings
Lei et al. [96]	Animal study	miRNA	The study showed that there was an expression of 136 miRNAs after 6 h from the injury, including: • Upregulation of 13 miRNAs with two folds • Downregulation of 14 miRNAs with two folds There was an expression of 118 miRNAs after 1 day from the injury, including: • Upregulation of 4 miRNAs with two folds • Downregulation of 23 miRNAs with two folds There was an expression of 149 miRNAs after 2 days from the injury, including: • Upregulation of 16 miRNAs with two folds • Downregulation of 11 miRNAs with two folds There was an expression of 203 miRNAs after 3 days from the injury, including: • Upregulation of 19 miRNAs with two folds • Downregulation of 5 miRNAs with two folds Also, the study showed that at all 4-time points following the injury, there was an upregulation of the expression of miR-21 According to microarray-based analysis, after TBI in the rat, the expression of miRNA in its cerebral cortex has demonstrated that there is miR-21 involvement in TBI intricate processes.
Hu et al. [97]	Animal study	miRNA	The study showed that: • There is a regulation of miRNA distinct sets at different times following the trauma. • For TBI progression assessment, the distinctive miRNA profiles at different times of post-CCI can be used as molecular signatures
Redell et al. [98]	Human study	miRNA	• The study aimed to assess the utility of plasma miRNAs in diagnosing severe and mild TBIs in the first 24 h after injury using quantitative RT-PCR. • Plasma miR-16, miR-92a, and miR-765 increased after severe TBI with AUC values of 0.89, 0.82, and 0.86, respectively. • Combining these miRNAs yielded high diagnostic accuracy with 100% specificity and 100% sensitivity. Only miR-92a and miR-16 plasma levels increased after mild TBI, with 0.78 and 0.82 AUC values, respectively.
Yang et al. [78]	Human study	miRNA	The study showed: • A significant increase in the serum concentrations of miR-191, miR-93, and miR-499 levels in TBI cases compared to the control group at all examined points of time. • The increase in these levels in severe TBI is more remarkable than mild or moderate TBI patients (*p* < 0.05). • Patients with poor outcomes had higher serum levels of miR-93, miR-499, and miR-191 compared to patients with a good outcome (*p* < 0.05).
Di Pietro et al. [99]	Human study	miRNA	The study showed that: • There was significant downregulation of miR-502 and miR-425-5p (*p* < 0.05) in mild TBI at early points of time, and they are ideal for mild TBI diagnosis. • There was significant upregulation of miR-335 and miR-21 (*p* < 0.01), and they are valid for use as diagnostic biomarkers for severe TBI. • miR-425-5p can predict the outcome of 6 months at T0-1 h and T4-12 h. On the other hand, miR-21 can predict the outcome at T4-12 h. • These miRNAs demonstrate that they are promising biomarkers for discrimination between mild TBI and severe TBI.
Bhomia et al. [100]	Human study	miRNA	In comparison with control samples, the data of real-time PCR showed that: • There was an upregulation of 39 miRNAs in mild and moderate TBI groups. • There was an upregulation of 37 miRNAs in a severe TBI group. • There was an upregulation of 33 miRNAs in the orthopedic injury group. In comparison between TBI groups and orthopedic injury: • There was an upregulation of 18 miRNAs in the mild and moderate TBI group • There was an upregulation of 20 miRNAs in the severe TBI group • Ten miRNA signatures were observed in both mild and moderate TBI (MMTBI) and severe TBI (STBI) groups. These miRNAs can be found in CSF and are valid to be used in the MTBI and STBI diagnosis.
Hicks et al. [101]	Human study	miRNA	The study showed that: • There was a detection of 214 miRNAs in the cerebrospinal fluid, and 135 miRNAs were detected in saliva. • There were parallel changes in 6 miRNAs in the saliva and cerebrospinal fluid (miR-29c-3p, miR-182-5p, mir-26b-5p, miR-30e-5p, miR-221-3p, miR-320c). • There were longitudinal trends expressed by three miRNAs in the cerebrospinal fluid and saliva following TBI, and they were associated with neuronal development. • There was a direct correlation between miR-320c concentration and attention difficulty.
Qin et al. [102]	Human study	miRNA	Results of miRNA microarray revealed that: • There was an upregulation of 65 miRNAs in mild TBI patients, 33 moderate TBI patients, and 16 severe TBI patients compared with healthy individuals. • There was a downregulation of 29 miRNAs in mild TBI patients, 27 miRNAs in moderate TBI patients, and six miRNAs in severe TBI patients. • There were 13 miRNAs (six downregulated and seven upregulated) detected in all TBI groups. The seven upregulated miRNAs indicated a good diagnostic accuracy. • Patients with severe TBI had higher miR-328-5p and miR-3195 levels in comparison with patients with mild and moderate TBI
Manek et al. [103]	Human study	Extracellular microvesicles/exosomes (MV/E)	• The study compared the microvesicles/exosomes (MV/E) in CSF samples of severe TBI patients and healthy controls. TBI increases the release of MV/E with elevated protein content. • Nanoparticle tracking analysis showed that the diameter of MV/E after TBI is less than the control, with a mode of 74-98 nm compared to a mode of 99-104 nm diameter, respectively. • The amount of MV/E in TBI CSF is greater than the control with values of 27.8–33.6 × 108/mL and 13.1–18.5 × 108/mL, respectively. • Using a targeted immunoblotting approach, MV/E of TBI CSF contained high concentrations of other TBI biomarkers, including UCH-L1, GFAP, and its breakdown products and αII-spectrin breakdown products. TBI CSF also contained synaptophysin, which is a presynaptic terminal protein, and an ALIX exosome marker. • A proteomic analysis of two TBI CSF and two control CSF samples, using nRPLC-tandem mass spectrometry, found 466 proteins in MV/E of TBI CSF and 91 proteins in control CSF
Ko et al. [104]	Human study	miRNA in brain-derived EVs	• The study assessed TBI and the brain state in mice using miRNA found in EVs via a microchip diagnostic technique. • Detecting the biomarker depended on combining machine learning algorithms with nanomagnetic isolation of EVs using specific surface markers and RNA sequencing arrays. • This technique achieved 99% accuracy in differentiating between injured and healthy groups. It identified injury intensity, detected previous injuries, and evaluated the time passed after injury. • This approach successfully identified various injuries in mice and different responses to these injuries, so it is a potential biomarker in detecting heterogeneous human TBIs.
Kuharić et al. [105]	Human study	EVs	The study showed that: • There was a detection of the highest range in EV concentration on the first day following the injury. • There was a significant increase in the size of EVs on days 4–7. • There was a detection of Flotillin-1 only in the cerebrospinal fluid in one-third of TBI patients. • There was a decrease in Arf6 concentrations and a delay in the increase of concentration of Rab7a in the cerebrospinal fluid. • There was a negative correlation between concentrations of Rab7a and Arf6 in the cerebrospinal fluid.
Goetzl et al. [106]	Human study	Neuron-derived exosomes (NDEs)	• The study compared plasma neuron-derived exosomes (NDEs) in acute and chronic mTBI patients and healthy controls, using anti-L1CAM antibody immunoabsorption. • NDE plasma levels normalized by CD81 maker decreased significantly in acute mTBI compared to the control group and did not change in chronic mTBI. • Many NDE neurofunctional protein levels significantly changed in acute mTBI. The plasma level of ras-related small GTPase 10 decreased, while many proteins increased, including annexin VII, UCH-L1, all spectrin fragments, claudin-5, and sodium-potassium-chloride cotransporter-1. Aquaporin 4 and synaptogyrin-3 increased in both acute and chronic mTBI. • Moreover, many neuropathologic proteins significantly increased in chronic mTBI, including CD81-normalized NDE levels of pathologic β-amyloid peptide 1-42, P-T181-tau, P-S396-tau, IL-6, and prion cellular protein (PRPc). IL-6 and PRPc also increased in acute mTBI. Detecting functional and pathologic proteins will help in understanding and predicting neurodegeneration associated with mild TBI.
Cheng et al. [107]	Human study	EVs	Real-time PCR analysis showed: • There was upregulation of 57 (15 genes, *p* < 0.05) genes in the patients of the emergency department. • There was upregulation of 56 (14 genes, *p* < 0.05) genes in the patients of the concussion clinic in comparison with the control group. • There was upregulation of three genes (CTSD, CDC2, and CSNK1A1) (*p* < 0.05) in the patients of the concussion clinic and emergency department.
Winston et al. [108]	Human study	Assessing neuronal- and astrocyte-derived exosomes	The study showed: • A detection of significantly higher plasma neuronal-derived exosomes and astrocyte-derived exosome levels of Aβ42 • There was significantly lower plasma neuronal-derived exosomes and astrocyte-derived exosome levels of the postsynaptic protein neurogranin (NRGN) in mild TBI patients compared to the control group. • Plasma neuronal- and astrocyte-derived exosome levels of neurofilament light (NFL), total tau, Aβ40, P-S396-tau, and P-T181-tau showed no difference between patients and control groups. • Plasma neuronal-derived exosome cargo proteins from samples of mild TBI were toxic to neuron-like recipient cells in vitro.
Puffer et al. [109]	Human study	EVs	The study showed that: • Plasma EVs contained GFAP in TBI patients suffering from alteration in consciousness ten times more than normal individuals or patients without altered consciousness. • There was an expression of 11 different miRNAs observed between these groups. These miRNAs are related to many cellular pathways, including organismal development, cellular development, and organismal injury.

#### Linkage Between EVs and Brain Injury

EVs may contribute to regeneration after stressful conditions. Oligodendrocytes, for example, release EVs that transfer antioxidant enzymes such as superoxide dismutase 1 and catalase to the neurons in response to oxidative stress [110]. Additionally, microglia-derived EVs containing miR-124 contribute to regeneration, suppress autophagy, decrease proinflammatory mediators, and increase anti-inflammatory mediators’ production [79].

As previously mentioned, TBI leads to abnormal folding and accumulation of proteins that contribute to neurodegenerative disease development and poor patients’ prognosis. These abnormal proteins stimulate a stress response called unfolded protein response (UPR). UPR originates from the endoplasmic reticulum (ER) to attenuate cellular stress and restore protein homeostasis (proteostasis). Proteostasis is achieved by decreasing the translation of new proteins and degrading the abnormal misfolded proteins [111]. UPR mediates proteostasis through three main pathways: PERK (protein kinase RNA–like endoplasmic reticulum kinase), ATF6 (activating transcription factor 6), and IRE1 (inositol requiring enzyme 1). Experimental studies reported that PERK pathway is activated after focal and diffuse TBI to maintain cell survival in such a stressful condition [112]. Other studies showed a promising therapeutic role of PERK pathway activation after TBI [113]. Additionally, ER stress and PERK pathway activation have been linked to increased EV expression to induce vascular calcification [114]. From this study, we hypothesize that ER stress after TBI may produce EVs with a different cargo that indicates UPR and correlates with a lower risk of neurodegenerative diseases. We suggest conducting experimental studies to examine this hypothesis and detect the EVs that reflect UPR after brain injury.

Autophagy is a homeostatic process in which there is a fusion of cytoplasm parts within multi-membraned vesicles termed autophagosomes, and then they undergo degradation by lysosomes [115, 116]. Recent studies have reported that there is persistent activation of the autophagy pathway after TBI, leading to neurological dysfunction including learning, sensory, motor, and memory impairments. These findings open the doors for finding a novel therapeutic intervention for TBI by the possibility of neuronal autophagy suppression [117–119].

It has been found that an increasing number of miRNAs can play a vital role in the modulation of autophagy major cascades, including autophagy induction, vesicle elongation, vesicle nucleation, autophagosome retrieval, autophagosome formation, autophagosome fusion to endosome/lysosome, autolysosome cargo degradation, and autolysosome formation, through targeting important autophagy molecular component or complexes [120]. For example, miR-21-5p, which was found to be upregulated in cultured neurons after *scratch injury* and in the brain cortex after TBI, has been reported to participate in autophagy regulation in several diseases in their pathological processes. It has been also observed that miR-21-5p targets Rab11a, which is known to be involved in both early and late stages of autophagy, suggesting that miR-21-5p expression may have an essential role in autophagy suppression [121]. Beside miR-21-5p, miR-124-3p may be involved in neuronal autophagy inhibition and nerve protection against injury as they increase in microglial exosomes following TBI. These findings provide a novel solution for nerve injury treatment following TBI through the introduction of microglial exosomes enriched with miR-124-3p [122].

#### Exosomes

According to many studies, exosomes can be an effective diagnostic and prognostic modality for TBI. Secretion of exosomes after TBI may serve to get rid of excess cellular products resulting from pathological processes or tissue damage induced by the disease [123, 124]. It was reported that exosomal RNA cargo could help identify patients susceptible to therapeutic failure and for intervention response monitoring. Changes in RNA profiles of exosomes may indicate disease recurrence and reflect the state of the originating cells [125]. A study that compared exosomes in the CSF of TBI patients and healthy individuals reported that the proteins in exosomes of TBI patients are five times more than exosomes obtained from the healthy population. These exosomes were suggested to have a potential role in cell-to-cell communication, and the detection of these exosomes may be beneficial for TBI diagnosis [23]. There are new techniques developed for monitoring exosomes like real-time PCR and microarray-based technologies and deep sequencing [126]. It was also found that there is a high increase in levels of exosomal GFAP in the blood of TBI patients with diffuse injury compared with those with focal injury. There was also a correlation between elevated levels of serum exosomal UCH-L1 and early mortality following TBI, which reflects the importance of this biomarker assessment [127]. Besides, increased exosomal levels of the neurofilament light (NFL) chain have been detected in patients with repetitive mild TBI and those with diffuse injury. NFL is an important marker for axonal injury resulting from degeneration or damages, including TBI (Table 2) [128].

Another subtype of EVs used for TBI diagnosis is microparticles (MPs) or microvesicles (MVs). MPs were reported to participate in the incidence and progression of injuries caused by TBI [129]. According to a study performed on mice, MPs may contribute to neuroinflammation following TBI as they can trigger microglial activation and many immunological responses [130]. MPs were found in high concentration in patients with severe TBI compared with healthy people [131]. These MPs are derived from leukocytes, endothelium, and platelets, and they have been suggested to help detect brain vascular injury as a result of TBI. Also, MPs carry proteins or antigens derived from brain cells, making them reliable TBI biomarkers [131–133]. Elevated levels of endothelial-derived MPs in the blood and CSF of TBI patients in the first days may indicate the level of vascular damage and a valuable tool for studying vascular and neuronal apoptosis [134].

#### Micro RNA

MiRNAs are a large group of small, endogenous, and highly conserved non-coding RNAs that modulate protein synthesis and gene expression at the post-transcriptional stage [135]. Initially, miRNAs are transcribed as long precursors in the nucleus called pri-miRNAs. Later, pri-miRNA is split by RNase Drosha to form many stem-loop products that are called pre-miRNAs [136]. Pre-miRNAs are transferred to the cytoplasm to be cleaved into the miRNA duplex by one of the ribonuclease family enzymes named Dicer. Half of this duplex transforms into mature miRNA while another half, called minor miRNA, will decay [137, 138].

Numerous miRNAs have been detected as gene modulators, and it has been shown that miRNAs regulate more than one-third of the human gene expression [138]. Several miRNAs are expressed in the cerebellum, hippocampus, midbrain, and frontal cortex [139–141]. Alteration of the profiles of miRNA expression is relevant to brain aging and brain growth [142]. Also, miRNA plays a significant role in synapse formation [143], differentiation, maturation, and neural genesis [141].

MiRNAs play a critical role in brain development and structuring the complicated neural network. However, miRNA abnormalities are relevant to many neurodegenerative diseases and several brain injuries such as TBI. Also, ischemic brain injuries lead to changes in miRNA levels, which further affects neuroprotective and pathophysiological processes. CNS comprises the highest miRNA concentration and diversity. It has been reported that 70% of miRNA molecules are expressed in the brain and spinal cord [144]. MiRNA expression develops throughout all the stages of neurodevelopment and differs across the regions of the brain [145]. MiRNAs also exhibit variations in intracellular localization within neurons [146]. Interestingly, MiRNA enrichment within the dendritic or axonal compartments implies that miRNAs might have special regulating functions in synapse formation, protein expression, and the construction of neuronal circuits [147]. Therefore, MiRNA expression is very critical to CNS function and development. Dysregulated miRNA levels have been correlated with impaired memory, learning, and cognition, in addition to various neuropsychiatric disorders [148]. These distinct characteristics resulted in a significant increase in miRNA utility as a biomarker for the pathology of CNS.

Investigations of miRNA release in TBI cases centered basically on serum miRNA variation among a few TBI cases and controls. Many studies demonstrated miRNA profile in the CSF and serum plasma after various severities of TBI at several points in time (Tables 2 and 3) [68, 78, 79, 98, 145, 149]. Redell and his colleagues showed that in the first 24 h of injury and by using the microarray method, there was a miR-92a and miR-16 downregulation in a lot of severe TBI cases and a miR-765 upregulation in other mild and severe cases [98]. Bhomia and colleagues evaluated miRNA profile in the serum plasma and CSF of patients who were classified into three distinct groups, mild and moderate TBI (MM-TBI), severe TBI, and patients who had orthopedic injuries with samples obtained in 44 h following the injury and compared with a control group of healthy individuals. In both MM-TBI and severe TBI groups, 18 and 20 miRNAs have been detected, of which ten existed at both TBI severities. Four out of these ten miRNAs have also been detected in the CSF [100].

**Table 3:** Diagnostic performance of the promising miRNAs identified in TBI patients

Biomarker	AUC	Sensitivity	Specificity	TBI severity	References
miR-16	0.89	100% for the three biomarkers combined	100% for the three biomarkers combined	Severe TBI	[98]
miR-92a	0.82				
miR-765	0.86				
miR-93	1.000	NR	NR	Mild TBI	[78]
miR-191	0.742				
miR-499	0.819				
miR-425-5p	1	NR	NR	Mild TBI	[99]
miR-502	1				
miR-21	0.961			Severe TBI	
miR-335	0.990				
miR-195	0.81	NR	NR	Mild to moderate TBI and severe TBI	[100]
miR-30d	0.75				
miR-451	0.82				
miR328	0.73				
miR-92a	0.86				
miR-486	0.81				
miR-505	0.82				
miR-362	0.79				
miR-151	0.66				
miR-20a	0.78				
miR-29c-3p	0.852	75% for the biomarkers combined	89% for the biomarkers combined	Mild TBI	[101]
miR-26b-5p					
miR-30e-5p					
miR-182-5P					
miR-320c					
miR-221-3p					
miR6867-5p	0.854	NR	NR	Mild TBI	[102]
miR-3665	0.877				
miR-328-5p	0.888				
miR762	0.916				
miR-3195	0.899				
miR-4669	0.907				
miR-2861	0.913				

Di Pietro and his colleagues investigated 754 miRNAs in the serum plasma of five severe TBI cases, five mild TBI cases, and five controls of healthy individuals at various points in time post-injury. Ten miRNAs were detected that changed with time. Two miRNA candidates (miR-502 and miR-425-5p) were validated earlier in thirty patients with TBI, and two miRNA candidates (miR-335 and miR-21) were validated for the control group of healthy individuals [99]. Finally, saliva has also been examined as a potential biomarker source for TBI. Changes in salivary miRNA have been detected in pediatrics with long-term concussion symptoms [150]. In patients with long post-concussive symptoms, five miRNAs (let-7a-3p, miR-133a-5p, miR-320c-1, miR-769-5p, and miR-320c-1) have been detected; 3 of them (miR-629, let-7b-5p, and miR-320c-1) were related to memorial complications, fatigue, and headache that developed within 4 weeks after head injury [101].

## Promising miRNA Candidates and Their Molecular Functions

In TBI patients, miR-320c is one of the most promising biomarker candidates. miR-320c has been altered in the serum plasma of adult patients with severe TBI and the saliva in pediatric patients with mild TBI [98, 150, 151]. In addition to this, this miRNA exhibits longitudinal variance in the patients’ CSF following severe TBI and estimates approximately the TBI symptom duration. Many previous studies have found that the levels of miR-320c in depressed individuals in their cerebral cortex change after suicide [152]. These outcomes are interesting given the relationship between prolonged concussion symptoms and the mode of depression. miR-320c role in neuroplasticity may facilitate this association [101].

Another intriguing candidate as a TBI biomarker is miR-92a. MiR-92a has been upregulated in the serum of cases with mild TBI and the CSF and serum plasma in cases with severe TBI [98, 100]. In acute and subacute post-injury periods, MiR-92a has been upregulated. Studies have demonstrated that the higher upregulated miR-92a suppresses angiogenesis following the ischemic events [153, 154]. Therefore, the miR-92a acute upregulation might be crucial in individuals with severe TBI in patients with ICH or neurovascular comorbidities.

**Fig. 2:**
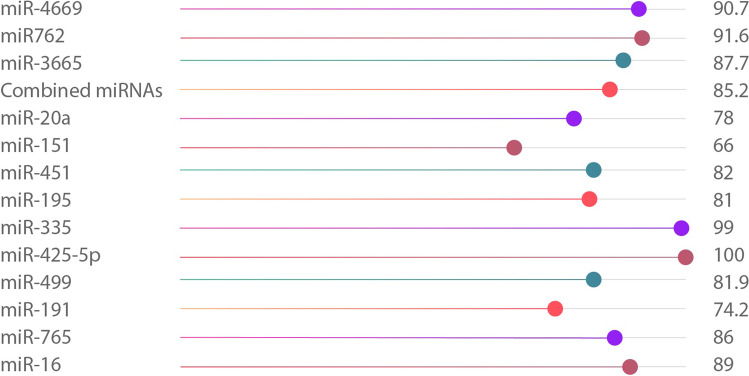
Bar chart showing area under the curve of diagnostic accuracy of miRNAs in TBI diagnosis. *Combined miRNAs = miR-29c-3p + miR-26b-5p + miR-30e-5p + miR-182-5P + miR-320c + miR-221-3p. Data are generated from the studies listed in Table 2

In multiple human TBI studies, various changes in the level of miR-30 were detected. MiR-30 expression changes have been observed in the serum plasma of cases with severe TBI within hours and days post-injury. In addition to serum plasma, changes in miR-30 have also been noted in CSF within weeks after TBI. It has been reported that there are changes in the miR-30 release in both blood and saliva [98–100, 148]. MiR-30 also has a magnificent role in neuroinflammation, and the pro-inflammatory cytokines negatively regulate it. A decreased miR-30 expression has been related to cell morphology changes, cell adherence loss, and increased cell migration because of the tight junction breakdown [155–157]. Thus, miR-30 can play a pivotal role in BBB maintenance. In Tables 2 and 3 and Fig. 2, we summarize data of the relevant studies conducted to evaluate the diagnostic performance of different types of MiRNAs in TBI patients.

## Conclusions

Due to the heterogeneous nature of TBI etiology, pathology, and clinical course, current conventional TBI biomarkers have several limitations in the clinical setting and TBI research. Neuroimaging by CT and MRI is limited to detecting gross head injuries without the minute neural injuries or the structural changes and their high costs. Fluid biomarkers seem reliable and more plausible for accurate correlation with neuronal, axonal, or glial cell injuries. However, current conventional fluid proteins might exist in small amounts that require sensitive assays and might even be undetectable.

Although exosomes and miRNA are minute structures whose levels significantly rise in TBI patients compared to healthy controls, they further demonstrated high accuracy in detecting TBI. For TBI patients with diffuse injuries, high levels of exosomal GFAP and NF light chains were detected. Several studies demonstrated miRNA profile changes in the CSF and serum plasma after various severities of TBI at several points in time. They showed the significant correlation between different levels of miRNA in the CSF and the blood of patients and the severity of the injury, which helps diagnose TBI patients and determines the severity of their injury. In addition, the isolation of exosomes and miRNAs is a more feasible process than the conventional fluid proteins. However, the current limitation for miRNA biomarkers is the variable expression in different individuals, particularly those with altered consciousness. This heterogeneity challenges determining the optimal miRNAs and cutoff values for TBI diagnosis and prognosis. Despite the current knowledge on the potential of exosomes and miRNAs, these biomarkers have not yet been optimized for clinical practice. However, according to the existing literature, they hold the promise as future biomarkers. Further research into exosomes and miRNAs in different TBI pathologies and at different time intervals is recommended to move them from the bedside to practice.

## Data Availability

Data sharing not applicable to this article as no datasets were generated or analyzed during the current study.
